# Effects of Extracts from Tiaozhi Granule and Its Components on Expression of Scavenger Receptor Class B Type I

**DOI:** 10.1155/2016/9238512

**Published:** 2016-12-06

**Authors:** Xiao Yu, Xiao-Dong Zhao, Rong-Qi Bao, Jia-Yu Yu, Guo-Xing Zhang, Jing-Wei Chen

**Affiliations:** ^1^Laboratory of Cancer Molecular Genetics, Medical College of Soochow University, Dushu Lake Campus, 199 Ren-Ai Road, Suzhou Industrial Park, Suzhou 215123, China; ^2^Department of Internal Medicine, The Affiliated Suzhou Chinese Traditional Medicine Hospital, Nanjing University of Chinese Medicine, 18 Yang-Su Road, Suzhou 215003, China; ^3^Department of Physiology, Medical College of Soochow University, Dushu Lake Campus, 199 Ren-Ai Road, Suzhou Industrial Park, Suzhou 215123, China

## Abstract

Sera from the rats with different drug treatments (atorvastatin, Tiaozhi granule, or its extracts) were collected. LO-2 cells or HepG2 cells were pretreated with different sera as the following groups randomly: (1) blank control group, (2) positive control group (atorvastatin group), (3) Tiaozhi granule water extract groups, (4) Tiaozhi granule alcohol extract groups, and (5) alcohol extracts for each component:* Pollen Typhae Angustifoliae*,* Curcuma longa* L., and* Rhizoma Alismatis*. LO-2 cells were cotransfected with plasmid carrying SR-BI and pRL-TK promoter genes. Promoter activity was measured by the luciferase reporter gene assay. The mRNA and protein expressions of SR-BI were examined using real-time PCR and western blot analyses. Our results show that promoter activity and mRNA and protein expression levels of the SR-BI were significantly upregulated by Tiaozhi granules alcohol or water extracts in a dose-dependent manner.* Pollen Typhae Angustifoliae* alcohol extract with a high dosage could also increase SR-BI activity and expression, but not the extracts from* Curcuma longa* L. and* Rhizoma Alismatis*. Both Tiaozhi granule alcohol and water extracts can upregulate SR-BI gene expression. Among the components,* Pollen Typhae Angustifoliae* are important for the regulatory effect coordinating with* Curcuma longa* L. and* Rhizoma Alismatis*.

## 1. Introduction

Atherosclerosis (AS) is one of the lipid metabolism disorders and chronic inflammatory diseases, which occurs within the large and/or medium-sized elastic vessels and intima or under intima of muscular arteries, characterized with progressive lipid deposition accompanied by fibrous tissue hyperplasia and inflammatory cell infiltration and forming atherosclerosis lesions or fiber lipid plaques, and eventually causes acute cardiovascular diseases by rupture of plaques [1–5]. Dyslipidemia is one of the most important risk factors for AS [6]. Hyperlipidemia (HLP) and AS are highly related to the morbidity and mortality of patients in the clinical investigations [7, 8]. A large number of epidemiological and genetic studies have shown that concentrations of low-density lipoprotein cholesterol (low density lipoprotein cholesterol, LDL-C) in plasma concentration are positively correlated with the occurrences of the AS, while concentrations of high-density lipoprotein cholesterol (high-density lipoprotein cholesterol, HDL-C) in plasma concentration are negatively correlated with the occurrences of AS [9, 10]. Previous studies focusing on lowering LDL and clinical treatment of lowering LDL have obtained certain achievements [11]. Researchers further began to identify the mechanisms responsible for the increase of LDL as the next targets for treatment of HLP and AS. In 1996, Acton et al. identified that SR-BI as a high-density lipoprotein receptor, which binds to HDL with a high affinity, is expressed primarily in liver and nonplacental steroidogenic tissues and mediates selective cholesterol uptake by a mechanism distinct from the classic LDL receptor pathway [12]. Reverse transportation of cholesterol (RCT) is one of the most important metabolic pathways in controlling cholesterol homeostasis. HDL is the main carrier of the body's RCT, promoting the transformation of cholesterol into bile acid and removing the cholesterol in the liver [13]. Recent studies revealed that rare variant in scavenger receptor BI raises HDL cholesterol, resulting in increase of the risk of coronary heart diseases [14]. Therefore, manipulation of SR-BI to adjust HDL metabolism for treatment HLP is a new strategy for the treatment of AS and cardiovascular diseases.

Wu Meng therapy has a long history for more than 2500 years in traditional Chinese medicine. Wan Da-Cheng, a grandmaster of Chinese Medicine, according to his long-term clinical experience, proposes that AS and HLP are inseparable, and the pathogenesis of HLP is mainly due to the blood stasis and congestion of arteries and veins by turbidity phlegm, advocating the concept that “ameliorating phlegm and stasis on the one hand, maintaining the liver normal function on the other hand.” He further developed the Tiaozhi granule to treat HLP patients in clinic, which is mainly composed of* Pollen Typhae Angustifoliae, Curcuma longa* L., and* Rhizoma Alismatis*. These components are also widely used in traditional Chinese medicine for the effects of antiatherosclerosis, anticancer, anti-inflammatory, and antioxidative activities [15–17]. Although various mechanisms have been identified for their therapeutic effects on treatment of clinical patients, the molecular mechanism related to the treatment of HLP and AS patients is still unclear. In the present study, we investigated the potential mechanisms of Tiaozhi granule on SR-BI expression in LO-2 cell line. In addition, we explored the possible effective components in Tiaozhi granule for the regulation of SR-RI expression.

## 2. Materials and Methods

### 2.1. Preparation of Extracts of Tiaozhi Granule and Its Components

#### 2.1.1. Alcohol Extract of Tiaozhi Granule

Three hundred grams of* Pollen Typhae Angustifoliae*, 300 g* Curcuma longa* L., and 300 g* Rhizoma Alismatis* powder were mixed and added to 0.9 L of 95% ethanol. The mixtures were heated for reflux extraction 2 times, and each cycle was for 2 hours. The solution was further filtered and condensed into thick paste, which was then boiled in a water bath with addition of 100 g hydroxypropyl cyclodextrin. Finally, 243.5 g extract powder was obtained containing 143.5 g alcohol extract of Tiaozhi granule.

#### 2.1.2. Water Extract of Tiaozhi Granule

Four hundred gams of* Pollen Typhae Angustifoliae,* 300 g* Curcuma longa* L., and 300 g* Rhizoma Alismatis* were sliced and added to 1.2 L of water. The mixtures were boiled for 2 hours. The solution was filtered and condensed. Finally, 203 g extract was obtained.

#### 2.1.3. Alcohol Extract of* Pollen Typhae Angustifoliae*,* Curcuma longa* L., and* Rhizoma Alismatis*

Three hundred grams of* Pollen Typhae Angustifoliae* powder was added to 0.3 L of 95% ethanol. The mixtures were heated for reflux extraction 2 times, and each cycle was for 2 hours. The solution was further filtered and condensed into thick paste, which was then boiled in a water bath with addition of 20 g hydroxypropyl cyclodextrin. Finally, 66.4 g extract powder was obtained containing 46.4 g alcohol extract of* Pollen Typhae Angustifoliae*.

Three hundred grams of* Curcuma longa* L. powder was added to 0.3 L of 95% ethanol. The mixtures were heated for reflux extraction 2 times, and each cycle was for 2 hours. The solution was further filtered and condensed into thick paste, which was then boiled in a water bath with addition of 20 g hydroxypropyl cyclodextrin. Finally, 51.8 g extract powder was obtained containing 31.8 g alcohol extract of* Curcuma longa* L.

Three hundred grams of* Rhizoma Alismatis* powder was added to 0.3 L of 95% ethanol. The mixtures were heated for reflux extraction 2 times, and each cycle was for 2 hours. The solution was further filtered and condensed into thick paste, which was then boiled in a water bath with addition of 20 g hydroxypropyl cyclodextrin. Finally, 52.6 g extract powder was obtained containing 32.6 g alcohol extract of* Rhizoma Alismatis*.

### 2.2. Serum Preparation for Cell Culture

Ten-week-old male Sprague-Dawley rats were purchased from Shanghai Laboratory Animal Center. Rats were housed under optimal conditions in the institutional animal facility. The experiments were performed in accordance with the National Institutes of Health Guidelines for the Use of Laboratory Animals (NIH, publication number 85–23, revised 1996.), which were approved by and performed according to guidelines for the care and use of animals established by Soochow University. Thirty-eight rats were randomly divided into 7 groups: (1) control group (*n* = 14), (2) positive control group (atorvastatin, *n* = 4), (3) water extract of Tiaozhi granule group (*n* = 4), (4) alcohol extract of Tiaozhi granule group (*n* = 4), (5) alcohol extract of* Pollen Typhae Angustifoliae* group (*n* = 4), (6) alcohol extract of* Curcuma longa* L. group (*n* = 4), and (7) alcohol extract of* Rhizoma Alismatis* group (*n* = 4). According to the surface area ratio of humans to rats, rats were administrated by gastric feeding with high dosage of each extract twice/day (high dosage is equivalent to twice dosage for clinical application). Dosages for atorvastatin, water extract of Tiaozhi granule, alcohol extract of Tiaozhi granule, alcohol extract of* Pollen Typhae Angustifoliae*, alcohol extract of* Curcuma longa* L., and alcohol extract of* Rhizoma Alismatis* are 0.9 mg/kg, 2.74 g/kg, 2.58 g/kg, 0.83 g/kg, 0.12 g/kg, and 0.59 g/kg, respectively. The dosages of each component applied in the present study are according to the clinical patient's dosages of Tiaozhi granule calculated by the ratio of surface area by humans to rats. All alcohol extracts were dissolved in 1% sodium carboxyl propyl cellulose. Three days after drugs administration, rats were sacrificed and blood was obtained from abdominal aorta. Sera were further separated from different groups and were mixed from 4 rats in the same group and inactivated and stored in −80°C.

### 2.3. Cell Culture

Human LO-2 hepatic cell line or HepG2 cell line was maintained in Dulbecco's modified Eagle's medium (DMEM) supplemented with 10% (v/v) fetal bovine serum. Cells were cultured with different concentrations of sera obtained from the rats fed with different drugs (high, 10%; medium, 5%; low, 2.5%). Additional sera from control rats were added to maintain 10% serum (v/v) for cell culture. Forty-eight hours later, cells were harvested for further analyses.

### 2.4. Transfection of LO-2 Cells and Luciferase Reporter Gene Assay

The reporter construct contains the sequence of the SR-BI gene from positions −1,200 to +2. The segment of interest was amplified by PCR and then cloned into a luciferase reporter vector (pSR-BI-LUC), as previously described [18]. Pure SR-BI recombinant plasmid of 0.5 *μ*g and pRL-TK plasmid 0.1 *μ*g were cotransfected into LO-2 cells using the Lipofectamine 2000 (Invitrogen, 11668-019, Shanghai, China). Forty-eight hours after transfection, cells were treated with different sera for 48 hours. Cells were then collected and lysed and luminescence intensity was measured. SR-BI promoter activity was normalized to the pRL-TK activity.

### 2.5. Real-Time PCR for SR-BI mRNA Expression

SR-BI mRNA expression was measured by RT-PCR as previously described [19]. In brief, total RNA was isolated from cells by guanidinium isothiocyanate-acid phenol extraction. One microgram of total RNA was used for reverse transcription and PCR assay. The primer pairs for SR-BI cDNA are 5′- TTG AAC TTC TGG GCA AAT G-3′ (forward) and 5′- TGG GGA TGC CTT CAA ACA C-3′ (reverse). The primer pairs for *β*-actin cDNA are 5′- GGA GAT TAC TGC CCT GGC TCC TA-3′ (forward) and 5′- GAC TCA TCG TAC TCC TGC TTG CTG-3′ (reverse). SR-BI mRNA expression was normalized to *β*-Actin mRNA.

### 2.6. Western Blot Assay for SR-BI Protein Expression

Expressions of SR-BI protein were measured by western blotting as our previous report [19]. Briefly, cells were harvested and lysates were resolved by 10% SDS-PAGE. Proteins were transferred to PVDF membranes (Hybond TM-ECL; Amersham Pharmacia Biotech, Inc.). The blots were incubated overnight with 1 : 1000 diluted primary antibodies, monoclonal anti-SR-BI and anti-GAPDH (Santa Cruz Biotech, Inc.), followed by incubation for 1 h with a secondary antibody (HRP-conjugated anti-rabbit IgG; 1 : 2000). Immunoreactive bands were then visualized using the enhanced chemiluminescence (ECL; Amersham Pharmacia Biotech) and analyzed by the NIH image software. Data were normalized to the expression of GAPDH.

### 2.7. Statistical Analysis

All data are presented as the mean ± SEM. Statistical significance between more than two groups was tested using one-way ANOVA followed by the Newman-Keel test. *P* values < 0.05 are considered statistically significant.

## 3. Results

### 3.1. Effects of Tiaozhi Granule Alcohol Extract on SR-BI Expression

**Figure 1:**
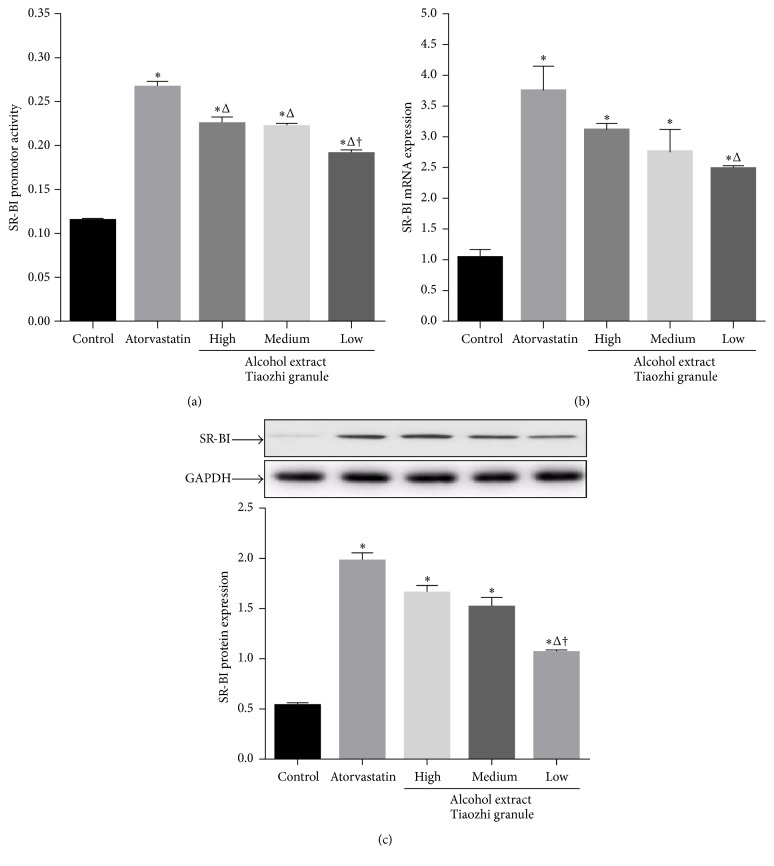
Effects of Tiaozhi granule alcohol extract on SR-BI expression in LO-2 cells. (a) Effect of Tiaozhi granule alcohol extract on SR-BI promoter activity (*n* = 12). (b) Effect of Tiaozhi granule alcohol extract on SR-BI mRNA expression (*n* = 6). (c) Effect of Tiaozhi granule alcohol extract on SR-BI protein expression (*n* = 6). The upper panels are representative blot of SR-BI and GAPDH; the lower panel is the densitometric analysis of SR-BI expression normalized to GAPDH. Data were presented as mean ± SEM. ^*∗*^ *P* < 0.05 compared with control group, ^Δ^ *P* < 0.05 compared with atorvastatin group, and ^†^ *P* < 0.05 compared with high and medium group.

After treatment of LO-2 cells with atorvastatin and alcohol extract from Tiaozhi granule, the SR-BI promoter activity was measured. Atorvastatin treatment significantly increased SR-BI activity compared with that of control group (*P* < 0.05). Alcohol extract of Tiaozhi granule with three different dosages also promoted SR-BI activity compared with control group (*P* < 0.05); however, it was markedly lower than the atorvastatin group (*P* < 0.05). In addition, low dosage of alcohol extract treatment showed a significantly lower SR-BI activity than that in the treatment groups with high and medium dosages of alcohol extracts from Tiaozhi granule (*P* < 0.05) (Figure 1(a)).

Consistent with the promoter activity results, mRNA expression levels in different treatment groups displayed similar patterns. Treatments with atorvastatin and alcohol extract of Tiaozhi granule with three different dosages significantly increased SR-BI mRNA expression compared with that of control group (*P* < 0.05). However, only the low dosage of alcohol extract treatment was markedly lower compared with the atorvastatin group (*P* < 0.05) (Figure 1(b)).

Besides the assays for mRNA expression, SR-BI protein expression levels in different treatment groups were also examined. Atorvastatin treatment significantly increased SR-BI protein expression compared with that of control group (*P* < 0.05). Furthermore, alcohol extracts of Tiaozhi granule with three different dosages also increased SR-BI protein expression. In addition, only low dosage of alcohol extract treatment was markedly lower than atorvastatin group and high and medium dosage groups of alcohol extracts from Tiaozhi granule (*P* < 0.05) (Figure 1(c)).

### 3.2. Effects of Tiaozhi Granule Water Extract on SR-BI Expression

**Figure 2:**
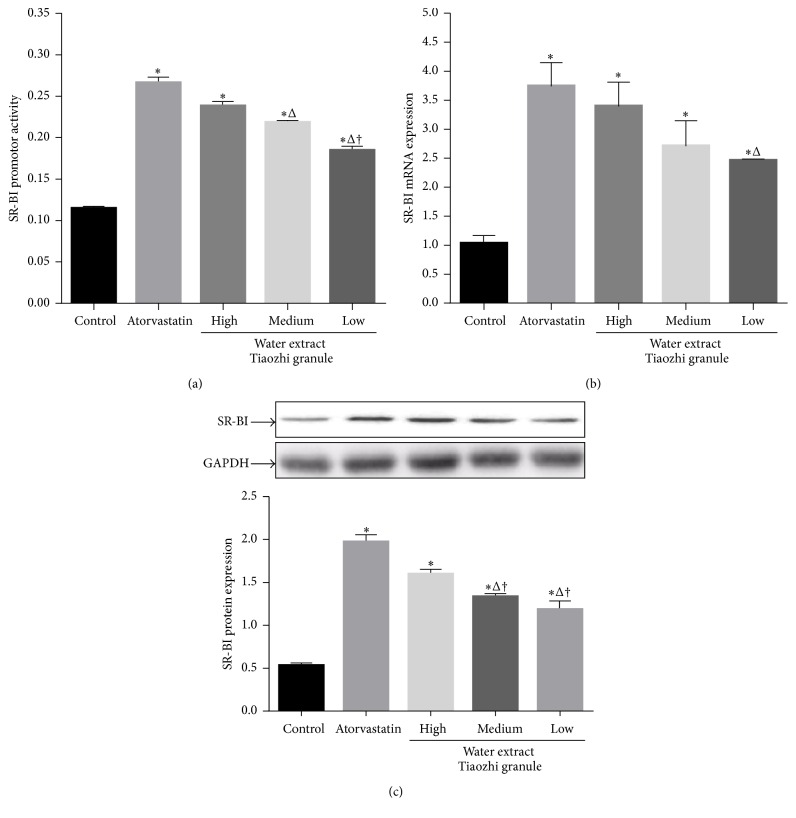
Effects of Tiaozhi granule water extract on SR-BI expression in LO-2 cells. (a) Effect of Tiaozhi granule water extract on SR-BI promoter activity (*n* = 12). (b) Effect of Tiaozhi granule water extract on SR-BI mRNA expression (*n* = 6). (c) Effect of Tiaozhi granule water extract on SR-BI protein expression (*n* = 6). The upper panels are representative blot of SR-BI and GAPDH; the lower panel is the densitometric analysis of SR-BI expression normalized to GAPDH. Data are presented as mean ± SEM. ^*∗*^ *P* < 0.05 compared with control group, ^Δ^ *P* < 0.05 compared with atorvastatin group, and ^†^ *P* < 0.05 compared with high and medium groups.

In order to exclude the possibility that the expression differences among the different groups are due to the different extract methods, effects of water extract of Tiaozhi granule were also investigated in LO-2 cells. Water extract of Tiaozhi granule with three different dosages also increased SR-BI activity; however, treatments with both low and medium dosages of water extracts were markedly lower than that of atorvastatin group (*P* < 0.05). In addition, treatment with the low dosage of water extract showed significantly lower SR-BI activity than that of treatment groups with high and medium dosages of water extracts from Tiaozhi granule (*P* < 0.05) (Figure 2(a)).

Water extracts of Tiaozhi granule with three different dosages also increased SR-BI mRNA expression; however, only low dosage of water extract treatment was markedly lower compared with that of atorvastatin group (*P* < 0.05) (Figure 2(b)).

Consistent with the mRNA expression levels, water extract of Tiaozhi granule with three different dosages also increased SR-BI protein expression. Furthermore, the expression levels in the treatment groups with both low and medium dosages of water extracts were markedly lower than atorvastatin group and the high dosage of water extract group (*P* < 0.05) (Figure 2(c)).

### 3.3. Effects of* Pollen Typhae Angustifoliae* Alcohol Extract on SR-BI Expression

**Figure 3:**
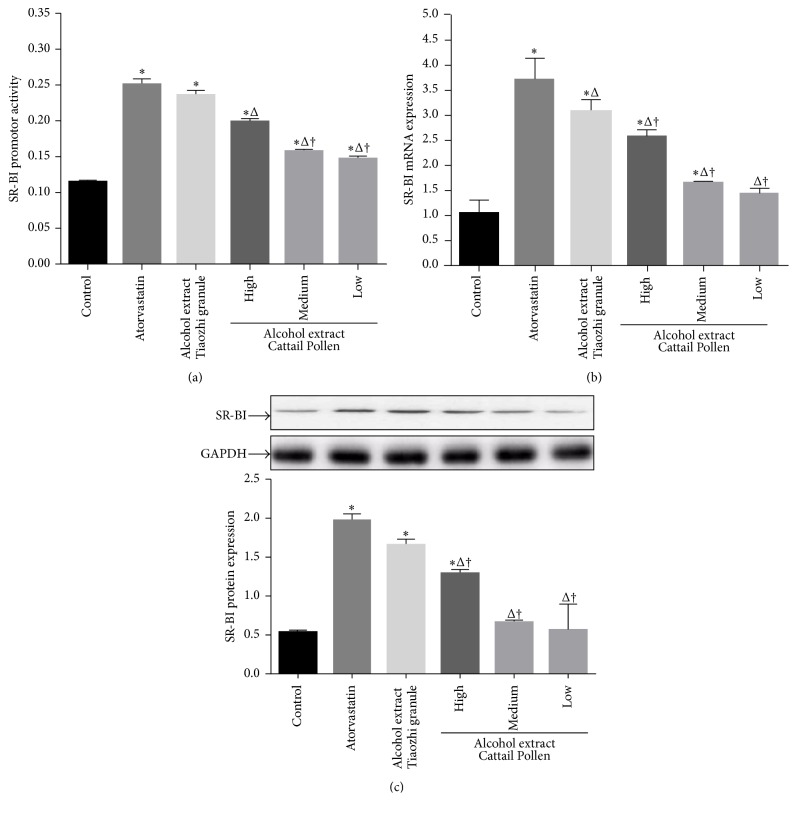
Effects of* Pollen Typhae Angustifoliae* alcohol extract on SR-BI expression in LO-2 cells. (a) Effect of* Pollen Typhae Angustifoliae* alcohol extract on SR-BI promoter activity (*n* = 12). (b) Effect of* Pollen Typhae Angustifoliae* alcohol extract on SR-BI mRNA expression (*n* = 6). (c) Effect of* Pollen Typhae Angustifoliae* alcohol extract on SR-BI protein expression (*n* = 6). The upper panels are representative blot of SR-BI and GAPDH; the lower panel is the densitometric analysis of SR-BI expression normalized to GAPDH. Data are presented as mean ± SEM. ^*∗*^ *P* < 0.05 compared with control group, ^Δ^ *P* < 0.05 compared with atorvastatin group, and ^†^ *P* < 0.05 compared with high dose of Tiaozhi granule alcohol extract group.

In order to further identify which component is important for the regulation of SR-BI expression, three components of Tiaozhi granule were investigated using the alcohol extract method in LO-2 cells. Alcohol extracts of* Pollen Typhae Angustifoliae* with three different dosages also increased SR-BI activity; however, the activity was markedly lower than atorvastatin treatment (*P* < 0.05). In addition, the low and medium dosages of alcohol extract treatments showed significant lower SR-BI activity than that of treatment with a high dosage of alcohol extract from Tiaozhi granule (*P* < 0.05) (Figure 3(a)).

Treatments with high and medium dosages of alcohol extracts from* Pollen Typhae Angustifoliae* also increased SR-BI mRNA expression; but the expression levels were all markedly lower than that of atorvastatin group and high dosage group of the alcohol extract from Tiaozhi granule (*P* < 0.05) (Figure 3(b)).

However, only high dosage of alcohol extract from* Pollen Typhae Angustifoliae* treatment increased SR-BI protein expression compared with control group (*P* < 0.05), and the expression level was markedly lower than that of treatments with atorvastatin and high dosage of alcohol extract from Tiaozhi granule (*P* < 0.05) (Figure 3(c)).

### 3.4. Effects of* Curcuma longa* L. Alcohol Extract on SR-BI Expression

**Figure 4:**
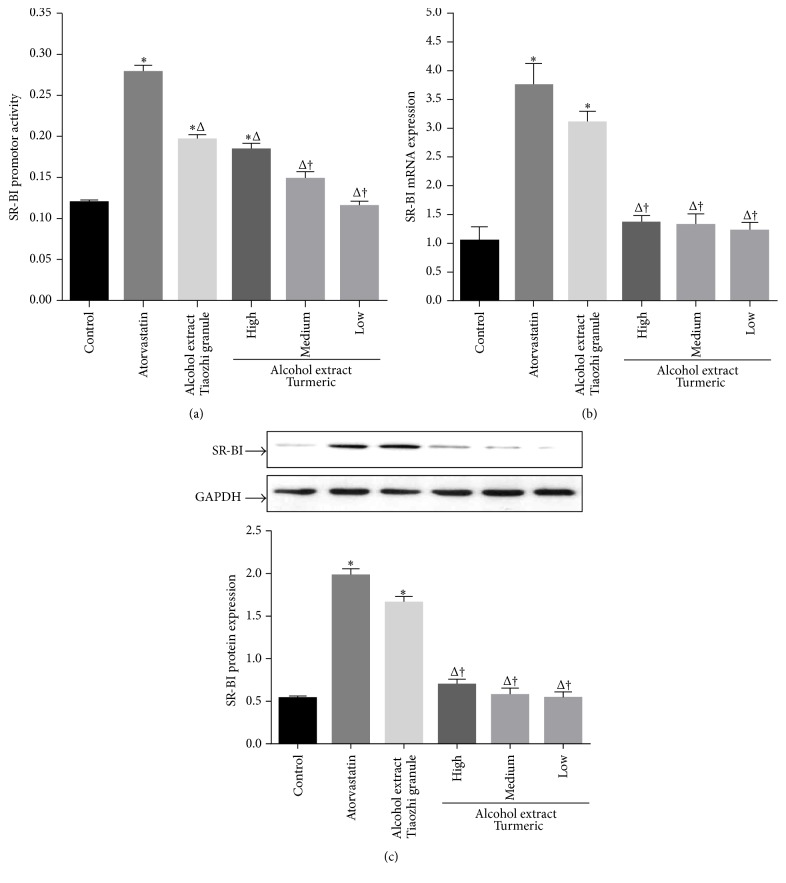
Effects of* Curcuma longa* L. alcohol extract on SR-BI expression in LO-2 cells. (a) Effect of* Curcuma longa* L. alcohol extract on SR-BI promoter activity (*n* = 12). (b) Effect of* Curcuma longa* L. alcohol extract on SR-BI mRNA expression (*n* = 6). (c) Effect of* Curcuma longa* L. extract on SR-BI protein expression (*n* = 6). The upper panels are representative blot of SR-BI and GAPDH; the lower panel is the densitometric analysis of SR-BI expression normalized to GAPDH. Data are presented as mean ± SEM. ^*∗*^ *P* < 0.05 compared with control group, ^Δ^ *P* < 0.05 compared with atorvastatin group, and ^†^ *P* < 0.05 compared with high dose of Tiaozhi granule alcohol extract group.

Only high dosage of alcohol extract from* Curcuma longa* L. can increase SR-BI promoter activity compared with the control group in LO-2 cells (*P* < 0.05, Figure 4(a)). However, it has no marked effects on SR-BI mRNA and protein expression with three different dosages (Figures 4(b) and 4(c)).

### 3.5. Effects of* Rhizoma Alismatis* Alcohol Extract on SR-BI Expression

**Figure 5:**
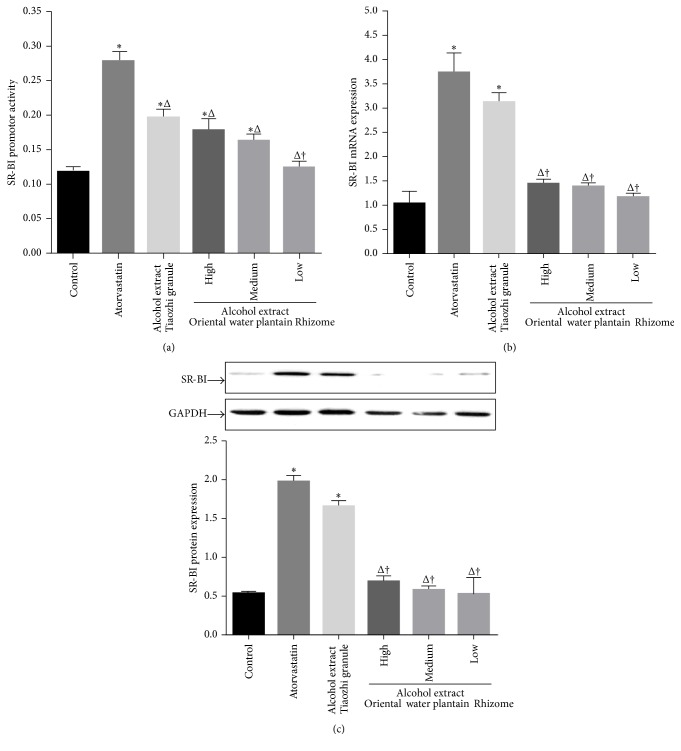
Effects of* Rhizoma Alismatis* alcohol extract on SR-BI expression in LO-2 cells. (a) Effect of* Rhizoma Alismatis* alcohol extract on SR-BI promoter activity (*n* = 12). (b) Effect of* Rhizoma Alismatis* alcohol extract on SR-BI mRNA expression (*n* = 6). (c) Effect of* Rhizoma Alismatis* alcohol extract on SR-BI protein expression (*n* = 6). The upper panels are representative blot of SR-BI and GAPDH; the lower panel is the densitometric analysis of SR-BI expression normalized to GAPDH. Data are presented as mean ± SEM. ^*∗*^ *P* < 0.05 compared with control group, ^Δ^ *P* < 0.05 compared with atorvastatin group, and ^†^ *P* < 0.05 compared with high dose of Tiaozhi granule alcohol extract group.

Both high and medium dosages of alcohol extract from* Rhizoma Alismatis* increased SR-BI promoter activity compared with control group in LO-2 cells (*P* < 0.05, Figure 5(a)), but it did not have marked effects on SR-BI mRNA and protein expression with three different dosages (Figures 5(b) and 5(c)).

### 3.6. Effects of Alcohol Extracts from Tiaozhi Granule and Its Components on SR-BI Expression in HepG2 Cells

**Figure 6:**
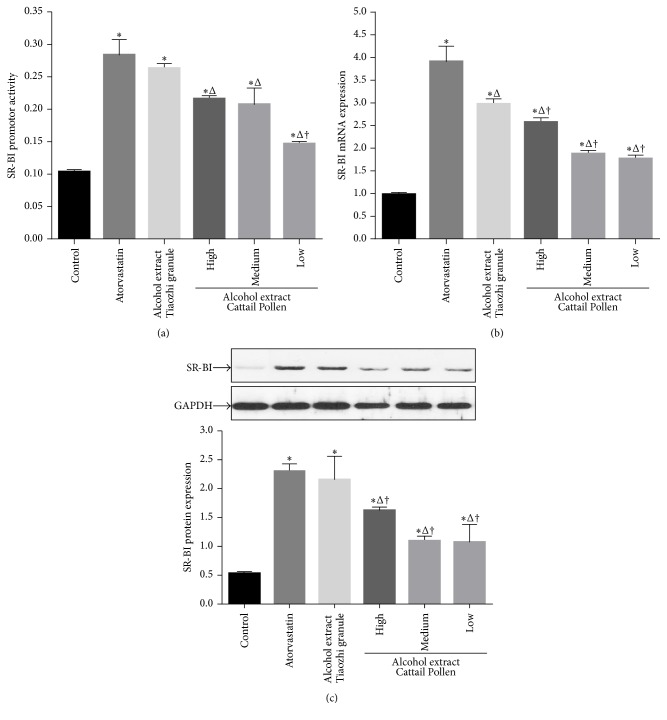
Effects of Tiaozhi granule alcohol extract and* Pollen Typhae Angustifoliae* alcohol extract on SR-BI expression in HepG2 cells. (a) Effects of Tiaozhi granule alcohol extract and* Pollen Typhae Angustifoliae* alcohol extract on SR-BI promoter activity (*n* = 12). (b) Effects of Tiaozhi granule alcohol extract and* Pollen Typhae Angustifoliae* alcohol extract on SR-BI mRNA expression (*n* = 6). (c) Effects of Tiaozhi granule alcohol extract and* Pollen Typhae Angustifoliae* alcohol extract on SR-BI protein expression (*n* = 6). The upper panels are representative blot of SR-BI and GAPDH; the lower panel is the densitometric analysis of SR-BI expression normalized to GAPDH. Data are presented as mean ± SEM. ^*∗*^ *P* < 0.05 compared with control group, ^Δ^ *P* < 0.05 compared with atorvastatin group, and ^†^ *P* < 0.05 compared with high dose of Tiaozhi granule alcohol extract group.

In order to further confirm the effects of Tiaozhi granule and its components on SR-BI expression, HepG2 cell line was also applied. Similar to the results in LO-2 cells, alcohol extracts from Tiaozhi granule and* Pollen Typhae Angustifoliae* dose-dependently increased SR-BI activity, mRNA, and protein expressions in HepG2 cells (*P* < 0.05) (Figure 6, only high dose data of Tiaozhi granule were shown). In addition, alcohol extracts from* Curcuma longa* L. and* Rhizoma Alismatis* did not increase SR-BI expression in HepG2 cells (data not shown).

## 4. Discussion

Tiaozhi granule is mainly composed of three herbs, with each having been widely used in traditional Chinese medicine for treatment of various diseases.* Pollen Typhae Angustifolia* is the traditional Chinese herbal medicine and widely used to treat the hemorrhagic diseases by both external and oral application. Its effective components-flavonoids and polysaccharides have been identified [20]. Qin and Sun have reported the immunosuppressive activity of* Pollen Typhae Angustifoliae* ethanol extract on the immune responses in mice [21]. Ohkura et al. observed that the activation of the intrinsic coagulation pathway by the acidic polysaccharide contributes to the external hemostatic property of* Pollen Typhae Angustifoliae*, and the components such as flavonoids that possess anticoagulant activity are causative agents when orally administered [22]. Later, anti-inflammatory activity and antioxidant potential of* Pollen Typhae Angustifoliae* were also reported [15, 23]. Recently,* Pollen Typhae Angustifolia* was reported to improve insulin resistance in the high-fat diet and low-dose streptozotocin-induced type 2 diabetic rats [24]. In the present study, we clearly show that* Pollen Typhae Angustifoliae* alcohol extract could increase SR-BI promotor activity, mRNA, and protein expression in LO-2 cell line, indicating that* Pollen Typhae Angustifolia* is an effective component in Tiaozhi granule, which may play an important role in the treatment of HLP, therefore, processing antiatherosclerotic effect.

*Curcuma longa* L., a herb widely farmed in Asia, is a primary constituent of traditional Chinese medicine [25] that has been used effectively for centuries to treat liver diseases in China. Curcumin, best known as a yellow pigment in* Curcuma longa* L., has been found to have antioxidant, anti-inflammatory, antihepatotoxic, and anticancer properties [16, 26, 27]. Recently, curcumin has been shown as a potential treatment for liver damage through manipulation of various signaling pathways. It can decrease the expression of proinflammatory mediators through downregulation of toll-like receptor 4 (TLR4) and TLR2 expression in CCl_4_-induced hepatic fibrosis [28] and damage [29]. Xu et al. also demonstrated that curcumin inhibits activation of hepatic stellate cells in vitro by reducing cell proliferation, inducing apoptosis, and suppressing ECM gene expression [30]. Based on the abovementioned literatures,* Curcuma longa* L. could protect liver from various damage and improve liver functions. Although in the present study we did not observe any effect of* Curcuma longa* L. on expression of SR-BI, we speculate that* Curcuma longa* L. may perform its function through other signal pathways or in assistance with other components to exert synergistic effects. Our results clearly support this issue that Tiaozhi granule has a stronger effect on regulation of SR-BI than any single component, even though* Pollen Typhae Angustifoliae* may play a pivotal role in the regulation of SR-BI.

*Rhizoma Alismatis* is also an important part of many prescriptions and has been commonly used for treating a wide range of ailments related to dysuria, edema, nephropathy, hyperlipidaemia, diabetes, and inflammation as well as tumor in clinical applications. Main chemical composition of* Rhizoma Alismatis* is the terpenoid including sesquiterpene, diterpene, and triterpene. The crude extracts and isolated compounds from* Rhizoma Alismatis* have diverse pharmacological activities including diuretic, nephroprotective, antihyperlipidemic, antiatherosclerotic, anticancer, anti-inflammatory, and antioxidative activities [17]. Recently, Li et al. demonstrated that triterpenes from the* Rhizoma Alismatis* extract can decrease serum lipid level in the high-fat-diet induced hyperlipidemia mice [31]. However, our present data did not show any effects of* Rhizoma Alismatis* on regulation of SR-BI expression. It may exert its function similar to* Curcuma longa* L. to synergize other components, or it may regulate lipid metabolism through lysophosphatidylcholine pathway [31]. A clinical trial is undergoing trying now, trying to confirm the effectiveness of* Rhizoma Alismatis* on improvement of blood lipid state [32].

In the present study, we did not investigate the molecular mechanism, by which the Tiaozhi granule and its component Pollen Typhae Angustifoliae upregulate SR-BI expression. It has been reported that inactivation of hepatic lipase by gene-directed targeting in mice results in upregulation of SR-BI expression in adrenal gland [33]. In addition, Spady et al. demonstrated that polyunsaturated fatty acids upregulate hepatic SR-BI expression [34]. We speculate that Tiaozhi granule and its component Pollen Typhae Angustifoliae upregulate SR-BI expression maybe through its antioxidant property to increase unsaturated fatty acids and/or through regulation of hepatic lipase signal pathways. Further study is needed to clarify this issue.

It should be noted that our present study only shows the effects of Tiaozhi granule and its components on SR-BI expression in vitro. Other molecular targets and whole body study are still needed to confirm the effectiveness of Tiaozhi granule and its components on HLP and AS patients, which may provide more solid evidence for the application of Tiaozhi granule and its components for clinic treatment.

In conclusion, our present data demonstrate that both water and alcohol extracts of Tiaozhi granule could regulate SR-BI expression. Among three components,* Pollen Typhae Angustifoliae* may play an essential role in the regulation SR-BI expression, while* Curcuma longa* L. and* Rhizoma Alismatis* may have synergistic effects on SR-BI expression.
